# Determinants of anxiety and depression in patients with cubital tunnel syndrome

**DOI:** 10.1186/s12888-020-02934-0

**Published:** 2020-11-17

**Authors:** Siming Jia, Xiaoying Shi, Guanglian Liu, Li Wang, Xiaoran Zhang, Xuelin Ma, Jia Li, Xinzhong Shao

**Affiliations:** 1 Department of Hand Surgery, The Third hospital of Hebei Medical University, Shijiazhuang, 050000 Hebei China; 2 Graduate School, Hebei Medical University, Shijiazhuang, 050000 Hebei China; 3 Department of Neurology, Hebei General Hospital, Shijiazhuang, 050000 Hebei China; 4 Department of Bone Surgery, Hebei Pingshan General Hospital, Shijiazhuang, 050000 Hebei China; 5 Department of Hand Surgery, Xuzhou Renci Hospital, Xuzhou, 221000 Jiangsu China

**Keywords:** Cubital tunnel syndrome, Anxiety, Depression, Neurophysiology, Hospital based study

## Abstract

**Background:**

The aim of this cross-sectional study to assess the proportions of anxiety and depression in patients with CuTS, and to explore the associated demographic and clinical features.

**Methods:**

From May 2011 to January 2017, 246 patients diagnosed with CuTS were recruited. The Hospital Anxiety and Depression Scale was used to assess the proportions of depression and anxiety. Patient demographic and clinical data were collected. Univariate analysis and multivariate regression were carried out to identify the variables that were independently associated with anxiety and depression.

**Results:**

The proportions of depression and anxiety were 17.9% (*n* = 44) and 14.2% (*n* = 35), respectively. Five patients had both possible/probable anxiety and depression. Logistic regression analysis revealed that diabetes mellitus was independently associated with depression; and the modified McGowan grade was independently associated with anxiety.

**Conclusions:**

In patients with CuTS, the proportions of depression and anxiety were 17.9% and 14.2%, respectively. Early screening for anxiety and depression is beneficial for patients with CuTS.

## Background

Cubital tunnel syndrome (CuTS) is the second most common peripheral nerve entrapment syndrome, caused by compression of the ulnar nerve within the cubital tunnel at the elbow [1–3]. Patients with CuTS are often troubled by symptoms like tingling and pain in the small finger and ulnar half of the ring finger [4]. As the condition progresses, muscular weakening and muscular atrophy will occur [5]. Owing to these symptoms, patients’ quality of life and the ability to continue employment may be impacted.

CuTS and depression are highly prevalent conditions, specifically among women [6]. In the general population, the global prevalence for major depressive disorder is 5%, whereas for anxiety disorders is 4% [7]. Mood disorders such as depression and anxiety increase the pain perceived and worsen the functional status [8]. Many published articles focus on disease progression, but few studies on patients’ mental status [9]. In a cross-sectional study, Van et al. [10] found patients with disorders on the upper extremity were more frequently reported anxiety and depression. They reported the proportions of depressive and anxiety disorders were 16 and 9% in patients with CuTS. Therefore, it is important to assess the mental status of patients with CuTS.

The aim of the study was to assess the proportions of anxiety and depression in patients with CuTS and to explore the associated demographic and clinical features.

## Methods

### Participants

**Fig. 1:**
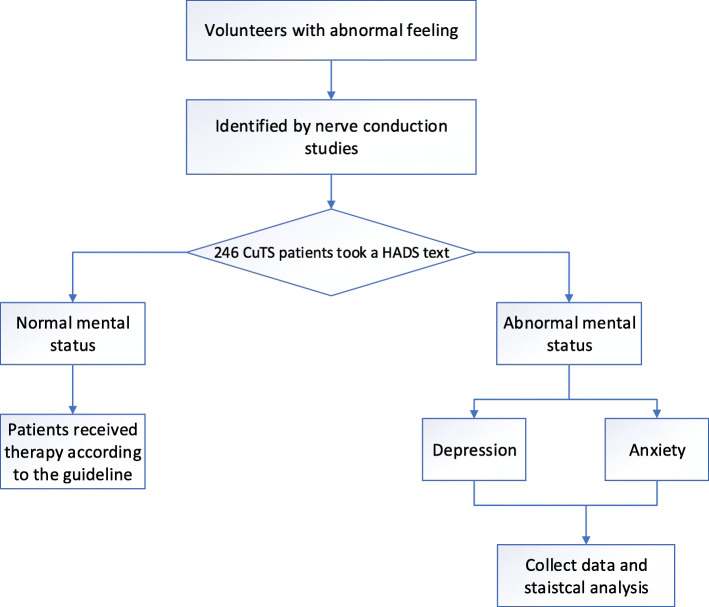
Flow chart of patients with CuTS who underwent HADS text

From May 2011 to January 2017, patients diagnosed with CuTS were recruited from the outpatient service of the hospital. The number of variable was used to calculate the optimal number of participants for this study. In this study, the sample size was typically expressed in terms of events per variable. An events per variable of 10 was widely used as the low limit for developing the models that predicted a binary outcome [11]. The sampling method was convenient sampling. The diagnosis of CuTS was made using a combination of clinical evaluation and nerve conduction study. Clinical assessments included the history of initial presence with intermittent paresthesias, numbness, and tingling in the small finger and ulnar half of the ring finger [4]. According to the guideline of the American Association of Electrodiagnostic Medicine [12], all patients underwent the nerve conduction study. Confirmatory criteria included: (1) motor nerve conduction velocity (MNCV) across the elbow less than 50 m/s; (2) an MNCV difference of greater than 10 m/s between the elbow segment and forearm segment; (3) a conduction block with compound muscle action potential decreased more than 20% (amplitude measured from the elbow to upper arm). Electrodiagnostic studies were performed by a specialist technician using a Dantec Keypoint Portable Nerve Conduction/ electromyography machine (Dantec Dynamics, Bristol, Bristol, UK) and reported by a consultant neurophysiologist. The specific process is shown in Fig. 1.

Patients were selected based on one of the following criteria: (1) patients with subjective symptoms, no matter the presence of intrinsic muscle atrophy or not; (2) electrodiagnostic evidence of CuTS; and (3) age > 18 years because the study just focused on adult patients. Patients with one of the following criteria were excluded: (1) age ≤ 18 years; (2) patients with other neuropathy confirmed electrophysiologically; (3) patients refused to attend the study; (4) patients who had undergone previous treatments, such as splinting, steroid injection, or cubital tunnel release; (4) a previous diagnosis of anxiety, depression, and other psychiatric disorder; (5) pregnant and lactating women because they were at high risks of depression and anxiety that distorted the assessments [13].

### Demographics and clinical evaluation

We used the self-administered questionnaire to assess the patients, which consisted of two parts. The first part included patients’ demographic data (age, gender, educational level, marital status, job status, and socioeconomic status). The second part was patients’ clinical data (hypertension, diabetes mellitus, tobacco use, alcohol use, history of cancer, and duration of symptoms). Patients were determined as older (> 50 years) and younger (≤ 50 years) adults. Educational level was registered as university degree, primary and middle degree, and illiterate degree. Marital status was coded as married, single (with or without cohabiting), widow, and divorced. Job status was registered as employed (in vacation or not) and unemployed (including students). Based on the classifying occupation, employed patients were classed into three groups: unskilled group, semi-skilled / skilled group and semi- professional/professional group. The criteria of classifying occupation were shown as follows: unskilled occupation: work not requiring education/training - eg: peon, watchman, domestic servant, laborer; semiskilled occupation: need some training to do the routine job efficiently - eg: laboratory attendant, library attendant; skilled occupation: long training in complicated work - eg: carpenter, mechanic, driver, telephone operator; semi-professional - high school teacher, lecturers in college, junior administrators, research assistants; professional- those involved in decision making, laying down policies and executing them - eg: doctors, senior administrative officers, senior lecturers, readers, professors, principals of colleges, advocates, engineers. The socioeconomic status was recorded based on the total amount of family income (US Dollar/per year) (high > 12, 735; medium = 4126 to 12, 735; low < 4126) [14]. The duration of symptoms was determined as long (> 2 years) and short (≤ 2 years) terms. Based on the standard of World Health Organization, alcohol use was defined as ≥60 g on one occasion in the past 30 days [15]. Patients were asked to complete the Quick Disabilities of the Arm, Shoulder, and Hand (QuickDASH) questionnaire to assess the hand function (0 = no disability, 100 = total disability) [16, 17]. It is a more quickly administered version of DASH, developed using Rasch analysis [17]. It has adequate convergent and discriminant validity, excellent internal consistency reliability with a Cronbach α of 0.89 in the primary care setting [18]. Based on the modified McGowan grade [10], the patients were classified into four groups (grade I: subjective symptoms, no abnormal objective findings; grade IIa: good intrinsic strength (4/5), no detectable muscle atrophy; grade IIb: fair intrinsic strength (3/5), detectable muscle atrophy; grade III: profound sensory and motor disturbances with marked intrinsic atrophy). We used the Chinese version Hospital Anxiety and Depression Scale (HADS) (≥11points, probable disorder; 8 to 10, possible cases; and ≤ 7, no case) to determine the anxiety and depression [19]. The HADS (21-item) contains anxiety sub-scale (HADS-A) and depression sub-scale (HADS-D) with each including 7 questions. We classified the patients as being depressed or anxious (present case, ≥8 points) or nondepressed/nonanxious (absent case, ≤7 points). The cut-off scores of depression and anxiety were 8 points. In China, the validated HADS is commonly used for interviewing participants. The Chinese version of HADS demonstrated the similar satisfactory linguistic equivalence, conceptual equivalence, and scale equivalence (concordance rates at the cutoff of 8 for anxiety and depression sub-scales were 89 and 87%, respectively; and at the cutoffs of 11 were 87 and 91%, respectively) compared with the English version [20].

### Data collection

Data was collected by a clinical psychologist (XS) who filled out the pre-coded structured questionnaire that comprised demographic and clinical data collected from the patients.

### Statistical analyses

The main outcomes were the proportions of anxiety and depression, as well as the associated factors. Associations between potential prognostic determinants and outcomes were examined using univariate logistic regression analysis. Predictors univariately associated with outcome (*p* < 0.05) were included in a multiple-predictor logistic regression model. Then multiple logistic regression (Backword-Wald) was carried out to identify the variables that were independently associated with anxiety and depression. Logistic regression was expressed as odds ratios with a 95% confidence interval (CI). Statistical analyses were performed using the Statistical Package for the Social Sciences (SPSS, version 25, Chicago, IL) for windows.

## Results

**Fig. 2:**
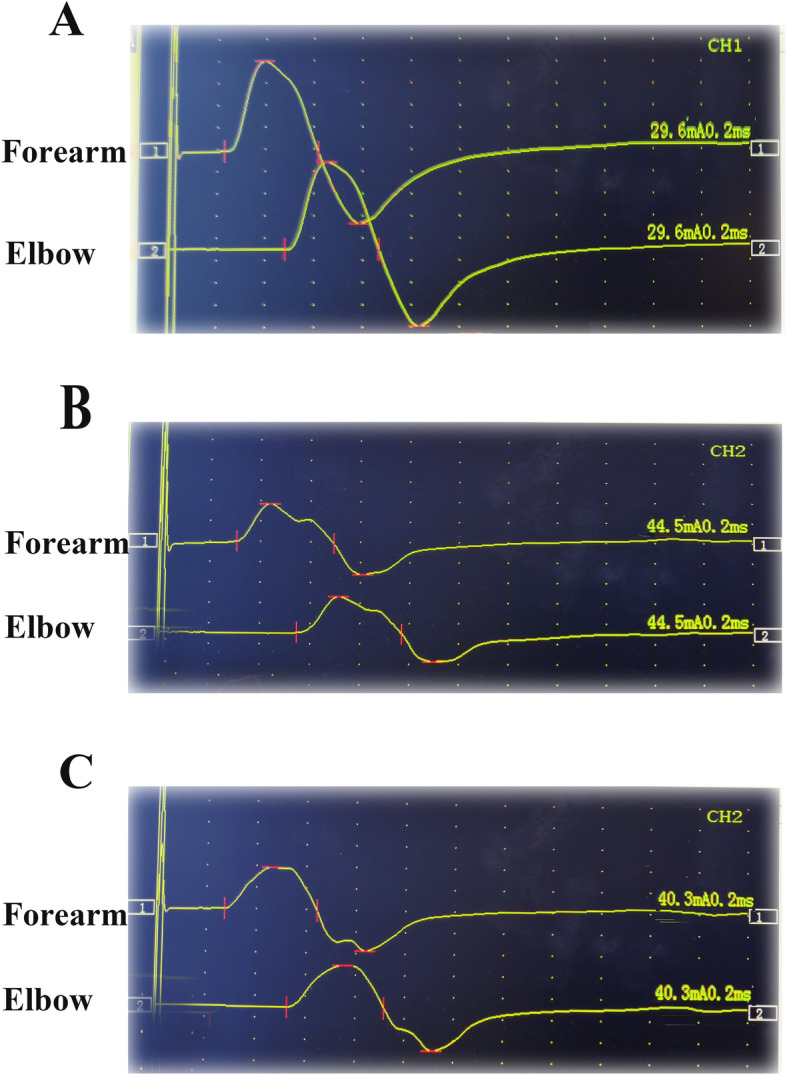
The picture of sample electrodiagnostic evidence of CuTS. **a** Patient with CuTS but without depression and anxiety symptoms. **b** Patient with both CuTS and depression symptoms. **c** Patient with both CuTS and anxiety symptoms

A total of 295 patients with typical electromyographic images of MNCV across the elbow (< 50 m/s) were recruited (Fig. 2). We excluded 49 patients because they refused to participate. Finally, 246 patients were included in this study with response rate of 83%. The mean age was 63.0 ± 3.8 years. There were 180 (73%) female patients; 166 (68%) patients had primary or middle education; 101 (41%) patients were in the medium socioeconomic level; 175 (71%) patients were married; 18 (7%) patients reported tobacco use; and 23 (9%) patients reported alcohol use. The most frequent medical comorbidities were diabetes mellitus (18%, *n* = 44) and hypertension (11%, *n* = 26). The average duration of symptoms was 23.9 ± 4.1 months. Based on modified McGowan grade, all patients were classified into grade I (*n* = 88), grade IIa (*n* = 45), grade IIb (*n* = 59), and grade III (*n* = 54) CuTS. The mean QuickDASH score was 41 ± 4. The demographic and clinical characteristics are summarized in Table 1.

**Table 1:** Demographic and clinical characteristics of study sample (*N* = 246)

Characteristics	
**Age (year)**	63.1 + 3.8
**Gender, n (%)**	
**Male**	66 (26.8)
**Female**	180 (73.2)
**Marital status, n (%)**	
**Married**	175 (71.1)
**Single**	1 (0.4)
**Divorced**	57 (23.1)
**Widowed**	13 (5.2)
**Education, n (%)**	
**University**	55 (22.3)
**Primary and middle**	166 (67.5)
**Illiterate**	25 (10.1)
**Job status, n (%)**	
**Unemployed (%)**	31 (12.6)
**Employed**	215 (87.4)
**Socioeconomic status, n (%)**	
**High**	24 (9.7)
**Medium**	101 (41.1)
**Low**	121 (49.2)
**Tobacco use, n (%)**	18 (7.3)
**Exceeding permitted alcohol limit, n (%)**	23 (9.3)
**Hypertension, n (%)**	44 (17.9)
**Diabetes mellitus, n (%)**	26 (10.6)
**History of cancer, n (%)**	1 (0.4)
**Duration of symptoms (months)**	23.9 ± 4.1
**QuickDASH score**	41 ± 4
**Modified McGowan grade, n (%)**	
**Grade I CTS**	88 (35.8)
**Grade IIa CTS**	45 (18.2)
**Grade IIb CTS**	59 (23.9)
**Grade III CTS**	54 (21.9)

The results of the HADS demonstrated that 44 (17.8%) patients presented with possible/probable depression, and 35 (14.2%) patients presented with possible/probable anxiety. Five patients had both possible/probable anxiety and depression.

The factors associated with depression were education (*p* = 0.001), job status (*p* = 0.003), Modified McGowan grade (*p* = 0.003) and diabetes mellitus (*p* < 0.001). The factors associated with anxiety were diabetes mellitus (*p* = 0.024), modified McGowan grade (*p* = 0.011), and hypertension (*p* = 0.024). The results of univariate logistic analysis are summarized in Table 2. Multivariable logistic analysis of univariate factors associated with depression and anxiety is shown in Table 3. Diabetes mellitus was independently associated with depression (*p* = 0.006). Modified McGowan grade was significantly associated with anxiety (*p* = 0.017).

**Table 2:** Demographic and clinical factors association with anxiety and depression among CuTS patients

	N	Depression			Anxiety		
	Present(*n* = 44)		Absent(*n* = 202)	*P* value	Present(*n* = 35)	Absent(*n* = 211)	*P* value
**Demographic characteristics**							
**Age, n (%)**							
**> 50 years old**	181	31 (70.5)	150 (74.3)		28 (80)	153 (72.5)	
**≦50 years old**	65	13 (29.5)	52 (25.7)	0.604	7 (20)	58 (27.5)	0.352
**Gender, n (%)**							
**Male**	66	16 (36.4)	50 (24.8)		5 (14.3)	61 (28.9)	
**Female**	180	28 (63.6)	152 (75.2)	0.115	30 (85.7)	150 (71.1)	0.071
**Marital status, n (%)**							
**Married**	175	30 (68.2)	145 (71.8)		23 (65.7)	152 (72.0)	
**Single**	1	0	1 (0.5)		0	1 (0.4)	
**Divorced**	57	10 (22.7)	47 (23.3)		9 (25.7)	47 (22.2)	
**Widowed**	13	4 (9.1)	9 (4.5)	0.624	3 (8.5)	10 (4.7)	0.733
**Education, n (%)**							
**University**	55	19 (43.2)	36 (17.8)		6 (17.1)	49 (23.2)	
**Primary and middle**	166	21 (47.7)	146 (71.8)		25 (71.4)	141 (66.8)	
**Illiterate**	25	4 (9.1)	21 (10.4)	0.001*	4 (11.4)	21 (9.9)	0.721
**Job status, n (%)**							
**Unemployed (%)**	31	12 (27.3)	19 (9.4)		5 (14.3)	26 (12.3)	
**Unskilled (%)**	**35**	9 (20.5)	26 (12.9)		4 (11.4)	31 (14.7)	
**Semiskilled/Skilled (%)**	**147**	19 (43.2)	128 (63.4)		19 (54.3)	128 (60.7)	
**Semi-professional/Professional (%)**	**33**	4 (9.1)	29 (14.4)	0.003*	7 (20)	26 (12.3)	0.606
**Socioeconomic status, n (%)**							
**High**	24	8 (18.2)	16 (7.9)		3 (8.6)	21 (10)	
**Medium**	101	15 (34.1)	86 (42.6)		10 (28.6)	91 (43.1)	
**Low**	121	21 (47.7)	100 (49.5)	0.103	22 (62.9)	99 (46.9)	0.206
**Clinical characteristics**							
**Diabetes mellitus, n (%)**	44	18 (40.9)	26 (12.9)	< 0.001*	11 (31.4)	33 (15.6)	0.024*
**Hypertension, n (%)**	26	7 (15.9)	19 (9.4)	0.204	8 (22.9)	18 (8.5)	0.011*
**Tobacco use, n (%)**	18	4 (9.1)	14 (6.9)	0.618	4 (11.4)	14 (6.6)	0.313
**Alcohol use, n (%)**	23	6 (13.6)	17 (8.4)	0.281	4 (11.4)	19 (9.0)	0.648
**History of cancer, n (%) #**	1	0	1 (0.4)	0.75	1 (2.8)	0	0.85
**Duration of symptoms, n (%)**							
**> 2 years**	124	25 (56.8)	99 (49)		18 (60)	106 (37.6)	
**≦2 years**	122	19 (43.2)	103 (51)	0.348	17((40)	105 (62.4)	0.896
**QuickDASH score**		41 ± 4	42 ± 3	0.061	40 ± 5	41 ± 3	0.082
**Modified McGowan grade, n (%)**							
**Grade I CuTS**	88	24 (54.5)	64 (33.7)		14 (40)	74 (35.1)	
**Grade IIa CuTS**	45	0	45 (22.3)		0	45 (21.3)	
**Grade IIb CuTS**	59	11 (25)	48 (23.8)		12 (34.3)	47 (22.3)	
**Grade III CuTS**	54	9 (20.5)	45 (22.3)	0.003*	9 (25.7)	45 (21.3)	0.021*

Chi-squared test was used, * *P* < *0*.05, # Fishers exact test was used

**Table 3:** Logistic regression for variables associated with anxiety and depression

	Variable	β	Odds ratio	95% CI	*p* value
**Depression**	Job status	−0.46	1.584	0.802–3.128	0.185
	Education	−0.365	0.694	0.209–2.306	0.551
	Modified McGowan grade	−0.304	0.738	0.457–1.193	0.215
	Diabetes Mellitus	1.717	5.570	1.650–18.806	0.006*
**Anxiety**	Modified McGowan grade	−0.533	0.587	0.378–0.911	0.017*
	Diebettes millitus	0.799	2.222	0.499–9.894	0.295
	Hypertension	1.378	3.965	0.804–19.568	0.091

Multivariable logistic analysis was used, * *P* < 0.05

Illiterate; unemployed and Grade III CTS were taken as a reference value in the calculation of Adjusted Odds Ratio for educational status, Job status and Modified McGowan grade

## Discussion

Many studies have shown that chronic diseases, such as diabetes and asthma, can cause mental health disorders after a prolonged period of time [21]. Beleckas et al. [13] found that the CuTS-related proportions of depressive and anxiety disorders were 16 and 9%, respectively, based on the National Institutes of Health developed the Patient-Reported Outcomes Measurement Information System. Anxiety may last for a long time before the medical intervention. Until now, few studies focus on the prevalence of depression and anxiety in patients with CuTS.

We found 18% of patients had significant symptoms of possible/probable depression disorder, and 14% had the symptoms of the possible/probable depressive disorder. Similarly, Belecka et al. [9] also found that patients with upper extremity conditions more frequently reported anxiety and depression than the general population did. Those findings are helpful for early psychiatric evaluation and identifying the presence of depression and anxiety in patients with CuTS.

Logistic regression analysis revealed that diabetes mellitus is independently associated with depression. Diabetes mellitus can cause small fiber neuropathy, which forms a significantly painful condition in a patient’s body [22]. Therefore, it is possible that the small fiber neuropathy worsens the symptoms of CuTS, and the cooperative effect increases the risk of depression.

In our study, the modified McGowan grade was independently associated with anxiety. We found a higher proportion of anxiety in patients with grade IIb (20%) and III (17%) CuTS, compared with that in patients with grade I (16%) and IIa (0) CuTS. Patients with anxiety tend to report worse hand function, and the worse hand function worsen their anxiety symptoms. This finding showed that worse anxiety is associated with higher symptom burden and worse physical functioning. Therefore, early intervention is beneficial to stop the progression of the disease for patients with mental health disorders.

Our study has limitations. The sample size is small, which affects the comprehension of the questionnaires presented to those participants. There is a bias that the HADS is a screening tool for assessing anxiety and depression rather than a clinical diagnostic tool. Our study is based on one time point and is thus cross-sectional. Therefore, we are unable to determine the causal relationships or establish the direction of associations.

## Conclusion

In patients with CuTS, the proportions of depression and anxiety were 18 and 14%, respectively. Early screening for anxiety and depression is beneficial for patients with CuTS.

## Data Availability

The datasets used and/or analysed during the current study available from the corresponding author on reasonable request.

## Abbreviations

CuTS: Cubital tunnel syndrome
QuickDASH: Quick disabilities of the arm, shoulder, and hand
HADS: Hospital anxiety and depression scale
CI: Confidence interval
SPSS: Statistical package for the social sciences
